# Supplementary material for: Co-Occurring Conduct Problems and Anxiety: Implications for the Functioning and Treatment of Youth with Oppositional Defiant Disorder
Source: Int J Environ Res Public Health. 2023 Feb 15;20(4):3405. doi: 10.3390/ijerph20043405 (PMC9962766; doi:10.3390/ijerph20043405)
Supplement: Supplementary file 1 [file ijerph-20-03405-s001.zip › ijerph-2207138-supplementary.pdf]

International Journal of
Environmental Research and Public Health

MDPI

Supplement

Method

Diagnostic interview

Separate interviews were conducted with the parents (ADIS-P) and the child (ADIS-C) by trained-to-criterion clinicians. Diagnoses were generated through a consensus based on information from both interviews. All interviews were videotaped and 20% were reviewed by a second clinician to compute Kappa coefficients. Using Cohen's Kappa, agreement on diagnoses was .77, .85, and .86, on primary, secondary, and tertiary diagnoses, respectively. An ODD diagnosis based the ADIS-C/P has been found to be reliable and valid [58].

Statistical Analysis

In the initial step of latent profile analysis (LPA), a one-class model is examined. In subsequent steps, profiles are added one at a time until there is no additional improvement to the fit of the model [59,60]. The optimal number of profiles was determined based on a variety of fit indices, the interpretability of each class on the basis of mean indicator scores, and our conceptual model. The Akaike Information Criterion (AIC) [50], Bayesian Information Criterion (BIC) [51], and sample size adjusted BIC (ABIC) [52], were used to assess the model's fit to the data, with lower values between successive models indicating better fit. The Bootstrap Likelihood Ratio Test (BLRT) was used to explore whether model fit significantly improved based on the addition of another class [59,60]. Specifically, BLRT examines whether the model with *k* classes provides a better fit to the data than the model with *k*-1 classes. A significant BLRT suggests that the current model has a better fit than the prior model with one fewer class.

In terms of fit indices, Monte Carlo simulations indicate that BIC and BLRT are the most robust indicators of model fit to the data and optimal number of classes to select [60] and were therefore relied on heavily when selecting the best-fitting model. Following the model selection, the latent class posterior probabilities for each observation obtained during this step-wise procedure were used to create a nominal value indicating the class to which each child most likely belonged.

Full Information Maximum Likelihood (FIML) estimation was used to address missing data. FIML uses all available data to estimate model parameters, but does not impute values. This approach generates smaller errors in parameter estimates and standard errors relative to other missing data strategies.

Supplement Tables

Table S1. Assessment measures by informant and internal consistency of the subscales.

Informant	Variable	Measurement	Cronbach's alpha/Kuder-Richardson reliability coefficient
Profile Classification			
Parents	Conduct Problems	BASC-2	Maternal 0.857; paternal 0.868
	Anxiety	BASC-2	Maternal 0.860; paternal 0.901
Teacher	Conduct Problems	BASC-2	0.904
	Anxiety	BASC-2	0.831
Child	ODD Symptoms	DBDRS	0.788
	Anxiety	BYI	0.915
External Validators			
Clinician	ODD Diagnosis	ADIS	-

	Anxiety Diagnosis	ADIS	-
	Global Functioning	CGAS	-
Parents	ODD Symptoms	DBDRS	Maternal 0.822; paternal 0.866
	CD Symptoms	DBDRS	Maternal 0.582; paternal 0.639
	Aggression	BASC-2	Maternal 0.837; paternal 0.830
	Emotional Self-Control	BASC-2	Maternal 0.778; paternal 0.775
	Emotional Lability	ERC	Maternal 0.775; paternal 0.822
	Proactive Aggression	CBRS	Maternal 0.797; paternal 0.793
	Reactive Aggression	CBRS	Maternal 0.578; paternal 0.734
	Executive Functioning	BRIEF	Maternal 0.952; paternal 0.968
Teacher	Aggression	BASC-2	0.921
	School Problems	BASC-2	0.677
	Learning Problems	BASC-2	0.846
	Study Skills	BASC-2	0.870
Child	Proactive Aggression	CBR	0.745
	Reactive Aggression	CBR	0.714
	Self-Concept	BYI	0.927

Note: ADIS = Anxiety Disorders Interview Schedule for DSM-IV, Child and Parent Versions; BASC-2 = Behavior Assessment System for Children, Second Edition; BRIEF = Behavior Rating Inventory of Executive Function; BYI = Beck Youth Inventory; CBRS = Child Behavior Rating Scale; CGAS = Child Global Assessment Scale; DBDRS = Disruptive Behavior Disorders Rating Scale; ERC = Emotion Regulation Checklist.

Table S2. Means, standard deviations, range, and zero-order correlations among classification variables.

**Variable**		**M**	**SD**	**Range**	**1**	**2**	**3**	**4**	**5**	**6**	**7**	**8**
Maternal-reported anxiety	Raw score	14.80	7.10	0-35	1							
	T-score	53.29	11.97	28-87								
Paternal-reported anxiety	Raw score	14.33	7.57	0-31	0.63**	1						
	T-score	52.48	12.80	28-80								
Teacher-reported anxiety	Raw score	3.40	3.23	0-12	0.24**	0.19*	1					
	T-score	49.82	11.03	38-79								
Self-reported anxiety	Raw score	12.71	9.47	0-52	0.08	0.10	-0.02	1				
	T-score	45.44	9.59	31-86								
Maternal-reported conduct problems	Raw score	12.53	4.74	4-24	0.07	-0.13	-0.09	0.12	1			
	T-score	71.42	12.87	48-103								
Paternal-reported conduct problems	Raw score	11.08	4.88	0-24	0.03	0.03	-0.13	0.18*	0.67**	1		
	T-score	67.42	13.34	37-103								
Teacher-reported conduct problems	Raw score	6.43	5.26	0-21	-0.15*	0.11	-0.06	0.01	0.30**	0.22**	1	
	T-score	51.98	21.21	0-93								
Self-reported conduct problems	Raw score	7.08	5.54	0-24	0.11	0.07	-0.05	0.15*	-0.00	-0.10	-0.08	1

*$p < .05$; **$p < .01$.

Table S3. Symptom scores across the four-profile model.

	**Profile 1** *n* = 42		**Profile 2** *n* = 33		Profile 3 *n* = 40		Profile 4 *n* = 19	
	**M**	**SD**	**M**	SD	M	SD	M	SD
Maternal-reported anxiety	7.73	5.66	18.79	7.27	16.26	8.00	15.79	12.03
Paternal-reported anxiety	6.31	5.96	24.32	5.52	14.32	6.05	13.34	5.00
Teacher-reported anxiety	1.96	3.63	3.28	3.85	4.59	6.28	3.73	6.97
Self-reported anxiety	11.18	8.96	12.63	11.27	9.84	10.71	17.71	17.45
Maternal-reported conduct problems	11.94	7.82	11.34	4.61	10.99	8.87	19.31	4.97
Paternal-reported conduct problems	10.61	6.60	11.92	5.49	8.23	6.16	18.30	8.24

Teacher-reported conduct problems	6.43	9.32	6.32	8.83	3.78	12.53	11.81	10.63
Self-reported conduct problems	8.34	6.74	6.30	5.96	4.97	8.06	9.15	14.16
